# Boosting Optical Nanocavity Coupling by Retardation Matching to Dark Modes

**DOI:** 10.1021/acsphotonics.2c01603

**Published:** 2023-01-11

**Authors:** Rohit Chikkaraddy, Junyang Huang, Dean Kos, Eoin Elliott, Marlous Kamp, Chenyang Guo, Jeremy J. Baumberg, Bart de Nijs

**Affiliations:** † NanoPhotonics Centre, Cavendish Laboratory, Department of Physics, University of Cambridge, JJ Thompson Avenue, CambridgeCB3 0HE, U.K.

**Keywords:** plasmonics, NPoM, impedance matching, dark modes, SERS

## Abstract

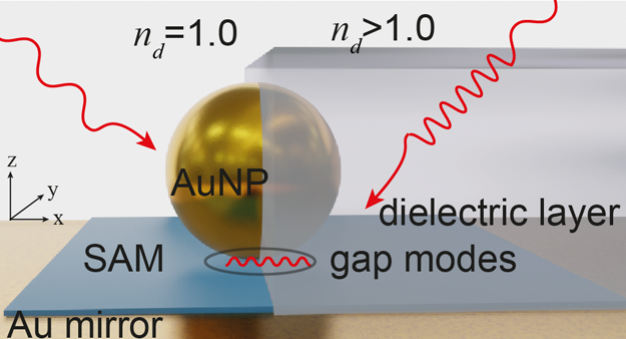

Plasmonic nanoantennas can focus light at nanometer length scales providing intense field enhancements. For the tightest optical confinements (0.5–5 nm) achieved in plasmonic gaps, the gap spacing, refractive index, and facet width play a dominant role in determining the optical properties making tuning through antenna shape challenging. We show here that controlling the surrounding refractive index instead allows both efficient frequency tuning and enhanced in-/output coupling through retardation matching as this allows dark modes to become optically active, improving widespread functionalities.

## Introduction

Plasmonic nanoconstructs with nanometer gaps confine light far below the diffraction limit, with potential applications in single-molecule sensing,^1^ adatom-catalysis,^2^ room-temperature quantum optics,^3−5^ and photon harvesting.^2,6,7^ Nanoconstructs which incorporate plasmonic nanogaps^8^ yield some of the highest^9^ and most reproducible^10,11^ field enhancements. Strong optical interactions with the metal surfaces slow down light in tightly confined modes, giving effective refractive indices *n*_eff_ ≫ *n*_g_, dependent on the gap thickness *d*, refractive index *n*_g_, and metal permittivity.^8^ Inconveniently for applications, the tightest confined modes emit at high angles (θ) to the nanogap normal, leading to poor in-/out coupling.^12^ As a result, net optical efficiencies of most nanocavity processes are ripe for enhancement,^21^ essential for transitioning nascent technologies into practical applications.

While plasmonic nanogaps support a few bright nanocavity modes, many modes are dark and only accessible via the near field.^13−20^ Making these bright and accessible at near normal incidence (θ = 0) would greatly improve optical access as it provides more operational frequencies and scattering angles, but how to do so is poorly understood and difficult to achieve. Plasmon resonances tune with the surrounding refractive index *n*_d_,^21−28^ although the antenna size, metal, and shape are more commonly employed to tune plasmon resonances instead as these effects have been well characterized and documented. Here, by mapping how *n*_d_ enhances specific plasmonic nanocavity mode coupling, we highlight improvements beyond simple wavelength shifts. We attribute this coupling enhancement to improved retardation matching between the slow light of the plasmon and retardation from the high refractive index surrounding medium. Finite-difference time-domain (FDTD) modeling matches comprehensive experimental characterization of plasmonic nanogap constructs coated in a range of dielectric media of different refractive indices. We show how modes shift across the visible and how dark antisymmetric modes become optically active. These amplified dark modes couple to the far field over a much wider angular range and are critically experimentally more accessible.

## Results and Discussion

To robustly form identical plasmonic nanogap constructs, a nanoparticle-on-mirror (NPoM) construct is used where a flat Au surface is coated with a molecular self-assembled monolayer to form a uniform spacer layer, here biphenyl-4-thiol (BPT) creating a ∼1.3 nm thick spacer.^29^ Colloidal *D* = 80 nm Au nanoparticles (AuNPs) are then deposited on top, forming a NPoM construct of high reproducibility.^11^ The optical hotspot in such nanogaps reaches intensity enhancements of 10^6^ and supports a set of optical modes dependent on the facet size, shape, polarization, and gap.^30^

**Figure 1:**
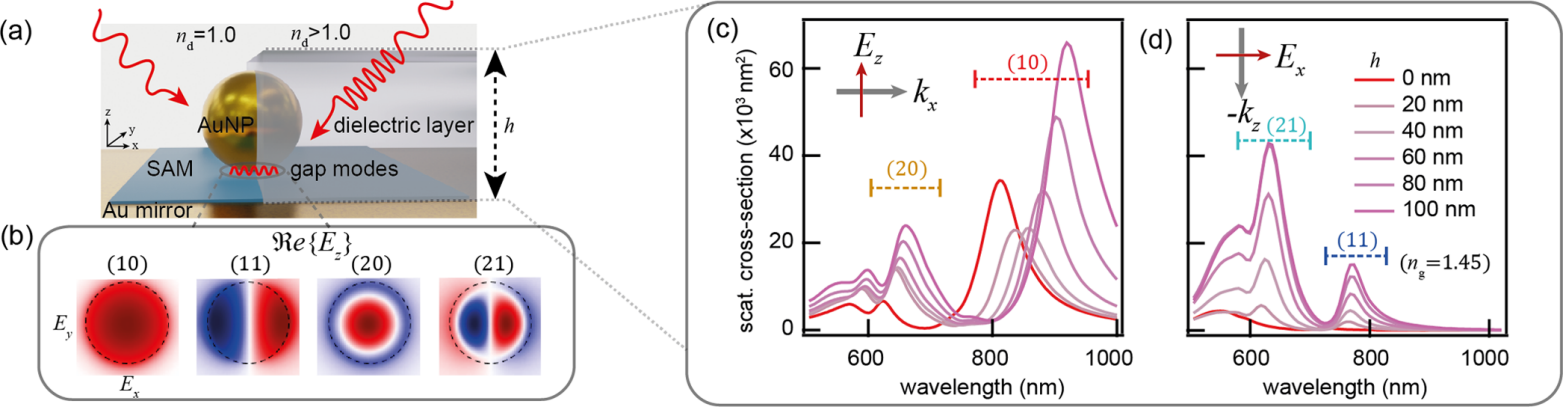
(a) Left: NPoM geometry in air (*n*_d_ = 1.0) on an Au surface with a thin dielectric spacer (1.3 nm, *n*_g_ = 1.45), right: NPoM embedded in the *n*_d_ = 1.5 dielectric layer of increasing height *h*. (b) FDTD-simulated near fields of four lowest energy modes in nanocavity, just above the mirror. (c,d) Effect of the dielectric layer height (*h*) on the gap modes under (c) high-angle and (d) normal-incidence illumination.

Full-wave FDTD simulations of these NPoMs truncate the AuNP to form a 20 nm circular bottom facet, capturing the faceting of colloidal AuNPs (Figure 1a: left).^31^ The plasmonic cavity formed between the AuNP and mirror supports a set of optical modes with the four lowest labeled (10, 11, 20, 21).^30^ These display characteristic field distributions (Figure 1b), with symmetric “even” modes (10, 20; denoted as *l*0) and antisymmetric “odd” modes (11, 21; denoted as *l*1).^30,32^ In air (*n*_d_ = 1), the even modes dominantly contribute to the far-field optical properties, while the odd modes are nonradiative (dark) and absent from the scattering spectrum. Introducing a high refractive index medium around the metal slows down the incident light, introducing a phase delay between antenna (NP top) and nanocavity (NP bottom), which matches the confined plasmons. Our simulations show that increasing the *n*_d_ = 1.5 dielectric film height (*h*) around the constructs shifts the even modes toward the infrared (Figure 1c), while the odd modes steadily become more radiative, as evidenced in scattering intensities (Figure 1d). The scattering strength of the (21) mode is comparable to the scattering intensities of the (20) mode for *h* > 80 nm, indicating efficient coupling to (21) at normal incidence in contrast with a high angle coupling to the (20) mode.

**Figure 2:**
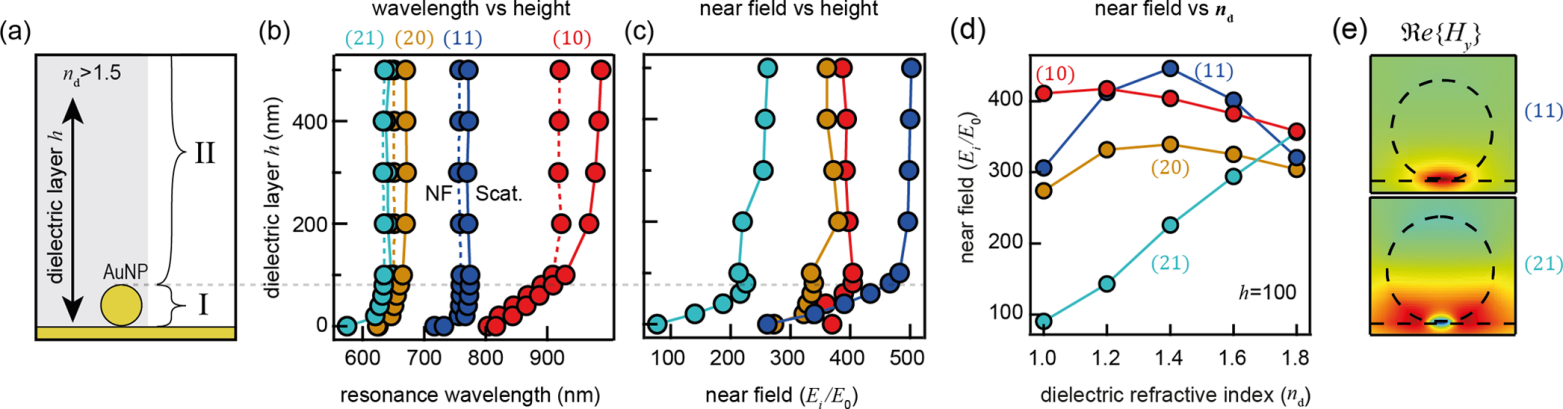
(a) Scheme depicting two regions of the dielectric layer height. (b) Tuning of resonance peak wavelengths extracted from scattering (solid lines) and near-field (dotted lines) spectra for each mode vs *h*. (c) Near-field enhancement (*E*_i_/*E*_0_) at spectral peaks of each mode vs *h*. (d) Near-field enhancement vs refractive index of the embedding dielectric material. (e) Optical field *H*_y_ (out of page) around NPoM for odd modes (11, 21) embedded in the dielectric coating of *n*_d_ = 1.5, *h* = 100 nm.

The NPoM’s optical properties change most strongly when a film intersects with the spill-out field of the gap and the near field of the nanoparticle, Figure 2a (region I), and saturates for *h* > *D* (region II). This is clearly observed in both near-field and scattering resonance wavelengths (Figure 2b,c). Upon embedding, the near field of odd modes is enhanced more than that of the even modes (Figure 2d), with (21) increasing by ∼250% and (11) by 100% compared to 30% for (20) and 10% for (10) modes (Figure S1a). Using fully embedded geometries (*h* = 100 nm) and instead increasing *n*_d_ show that the near field of the radiative (10) mode decreases (Figure 2d), primarily as its red-shifting resonance is less well confined within the nanogap. The near field of the nonradiative (21) mode however greatly increases, becoming comparable to the fundamental (10) mode at *n*_d_ = 1.8. For (11, 20), strongest near fields are observed near *n* = 1.4 from the two competing effects. The larger field spill-out of the AuNP facet for odd modes is visualized from the magnetic field,  (Figure 2e). The resonance shifts, increase in scattering intensities, and near-field enhancements clearly highlight the importance of the refractive index from the surrounding medium in determining the optical properties of plasmonic nanogap constructs.

**Figure 3:**
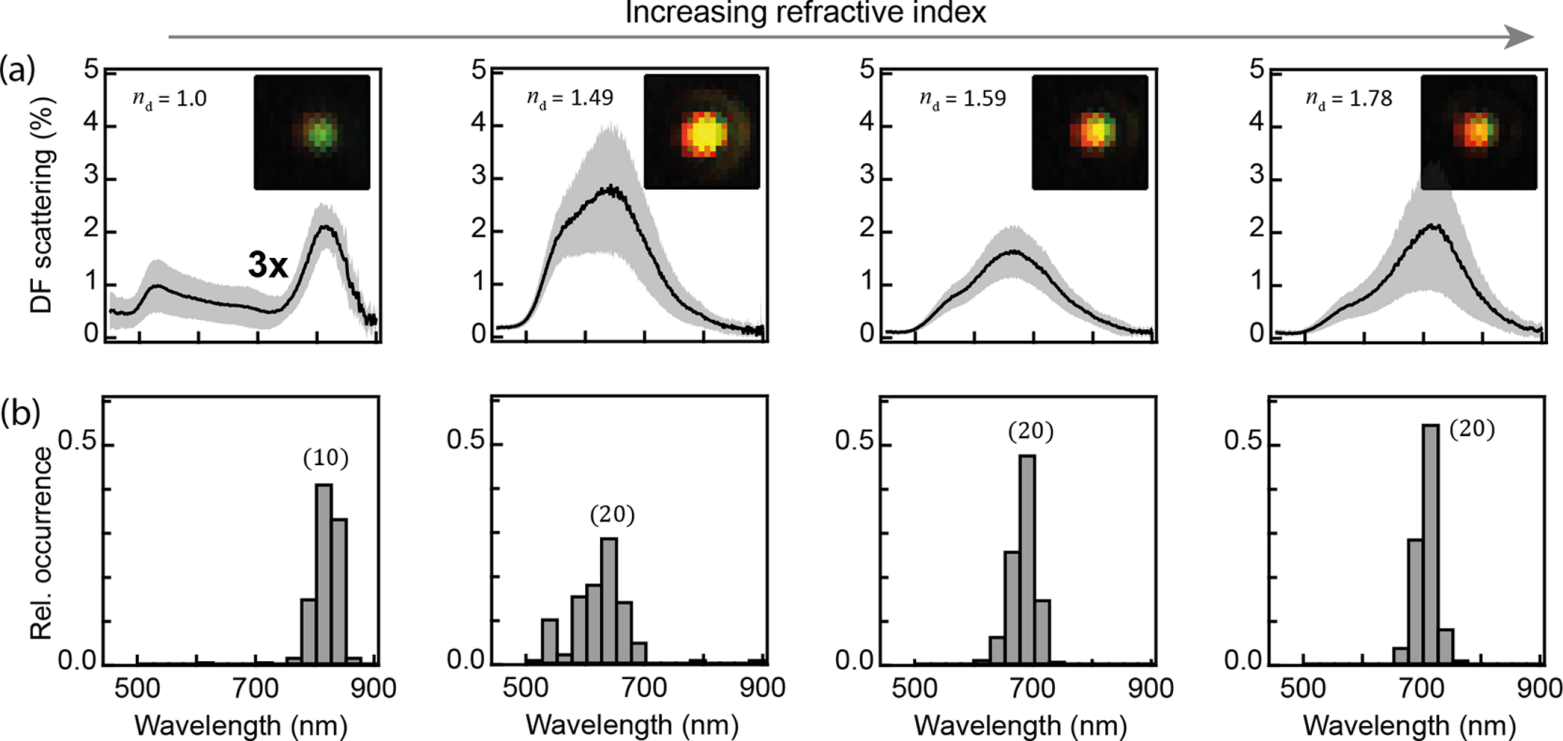
(a) Experimental DF scattering spectra for NPoMs (*D* = 80, 1.3 nm spacer) inside progressively higher refractive index coatings (*n*_d_ = 1.0, 1.49, 1.59, 1.78), note *n*_d_ = 1.0 multiplied 3× for visibility. Black line indicates the average of 1550, 313, 438, and 2235 NPoMs, respectively, and gray color indicates 50% confidence interval. Insets: average DF scattering images. (b) Relative occurrence of the main DF visible spectral peak, which red-shifts with increasing refractive index. The (10) mode at *n*_d_ = 1.0 red-shifts outside the detection range (>900 nm) for *n*_d_ ≥ 1.2.

To evidence these changes experimentally, NPoM geometries are prepared with a range of different refractive index coatings (Figure 3). Dielectric layers 100 nm thick with a refractive index *n*_d_ = 1.49, 1.59, or 1.78 are spin-coated onto the NPoMs described above. The average dark-field (DF) spectra (Figure 3a) of many hundred NPoMs show how the plasmonic modes evolve with increasing refractive index. The scattering intensity from polymer-coated NPoM nanoantennas is over 3-fold brighter than NPoMs in air (*n* = 1), attributed to improved in-/out coupling of light (see Supporting Information Note S4). Upon coating, the dominant (10) mode visible at 810 nm in air disappears (red-shifting out of the detection range), and higher-order modes red-shift and increase in intensity. Extracting the dominant peak position for each refractive index (Figure 3b) shows higher-order modes at 650, 695, and 710 nm for *n* = 1.49, 1.59, and 1.78, respectively. To test reproducibility, three more repeats of *n* = 1.49 poly(methyl methacrylate) (PMMA)-coated NPoMs are analyzed, each showing excellent agreement in average DF position and scattering intensity (Figure S2).

**Figure 4:**
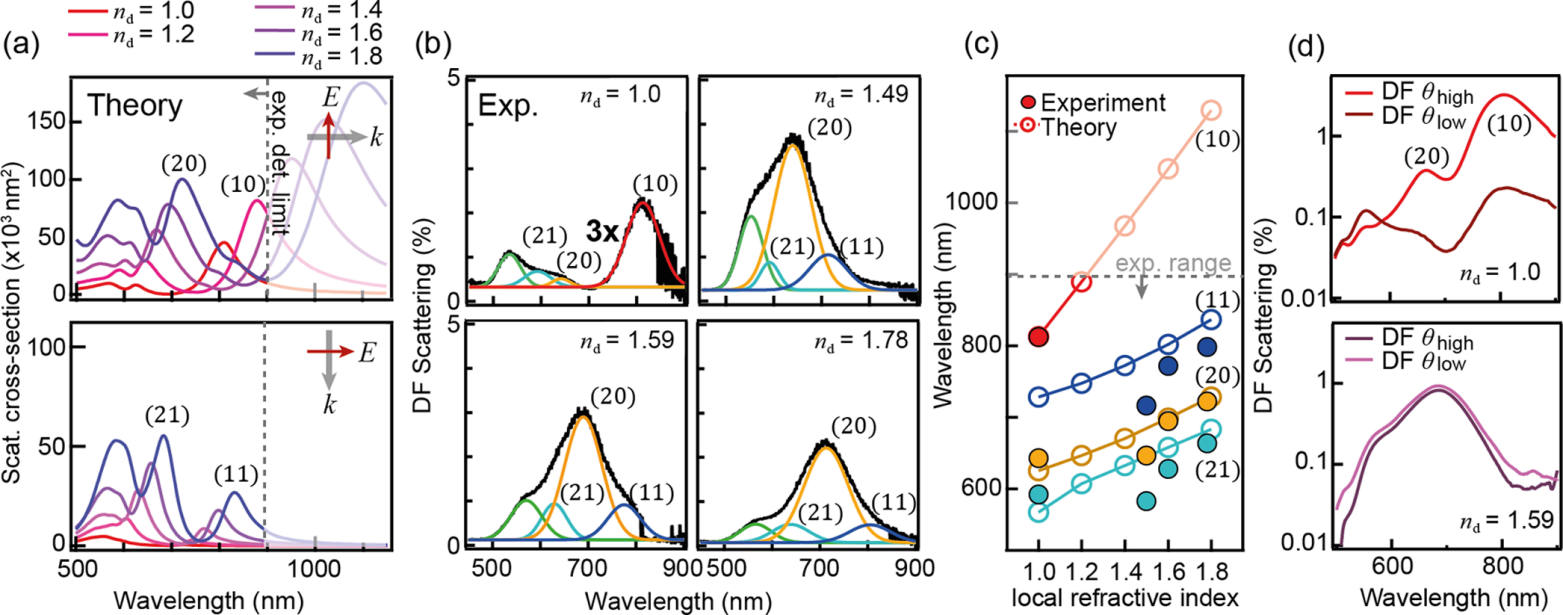
(a) Calculated DF scattering spectra in dielectric media of refractive indices *n*_d_ = 1.0–1.8 showing increasing red shifts. Simulations use two different illumination conditions: (top) high angle with a *E*⊥ mirror surface and (bottom) normal illumination. Insets show *E*, *k* directions. (b) Experimental DF scattering spectra for *n*_d_ = 1.0, 1.49, 1.59, 1.78 using unpolarized illumination at high angles. Spectra separated into optical modes using multi-Gaussian fit, assigned to different modes. (c) Extracted (solid) and modeled (open) peak positions vs surrounding refractive index. (d) Angle-resolved DF scattering spectroscopy of NPoMs shows that high angles dominate for *n*_d_ = 1.0 (top), but low angles dominate for *n*_d_ = 1.59 (bottom).

Modeling the effect of the refractive index on the fully embedded NPoMs (*h* > 100 nm) reproduces the red shifts and a rise in scattering intensity of all modes with increasing *n*_d_ (Figure 4a). Comparing the simulations with experimental DF spectra (Figure 4b) enables assignment of the dominant modes, (10): red, (20): yellow, with the satellite peaks tentatively assigned to (11, 21). The peak positions of these modes are in agreement with predictions (Figure 4c), except for *n*_d_ = 1.49, where all peaks are blue-shifted (possibly due to coating morphology under AuNPs). We note that simulations here also do not capture variations in nanoparticle facet shape, which further breaks the degeneracy of (*l*1) modes.^30,33^ While residual citrate molecules and a thin layer of water might remain coating the AuNPs (after thorough rinsing of the NPoMs), which may increase the refractive index, the effect on the modes is minimal because this coating would be of sub-nanometer.

The simulations predict a significant increase in scattering intensity from the initially dark odd modes, which emerge as satellite peaks in the DF spectra (Figure 4b,c). The modes can be distinguished by characterizing their different out-coupling angles. Even modes (with vertical dipoles) should emit at high angles and dominate radiation for *n*_d_ = 1.0 when separating high-angle (emitted flux at θ_high_ = 55–64°) from low-angle (θ_low_ = 0–10°) scattering in *k*-space spectroscopy (Figures 4d, top, and S4a).^34^ In contrast, for *n*_d_ = 1.59, nearly equal radiant intensities are simulated for low and high collection angles (Figures 4d, bottom, and S4b). This confirms that out-coupling from high-index-coated NPoMs is at lower angles, yielding high collection efficiencies even in low numerical aperture systems.

**Figure 5:**
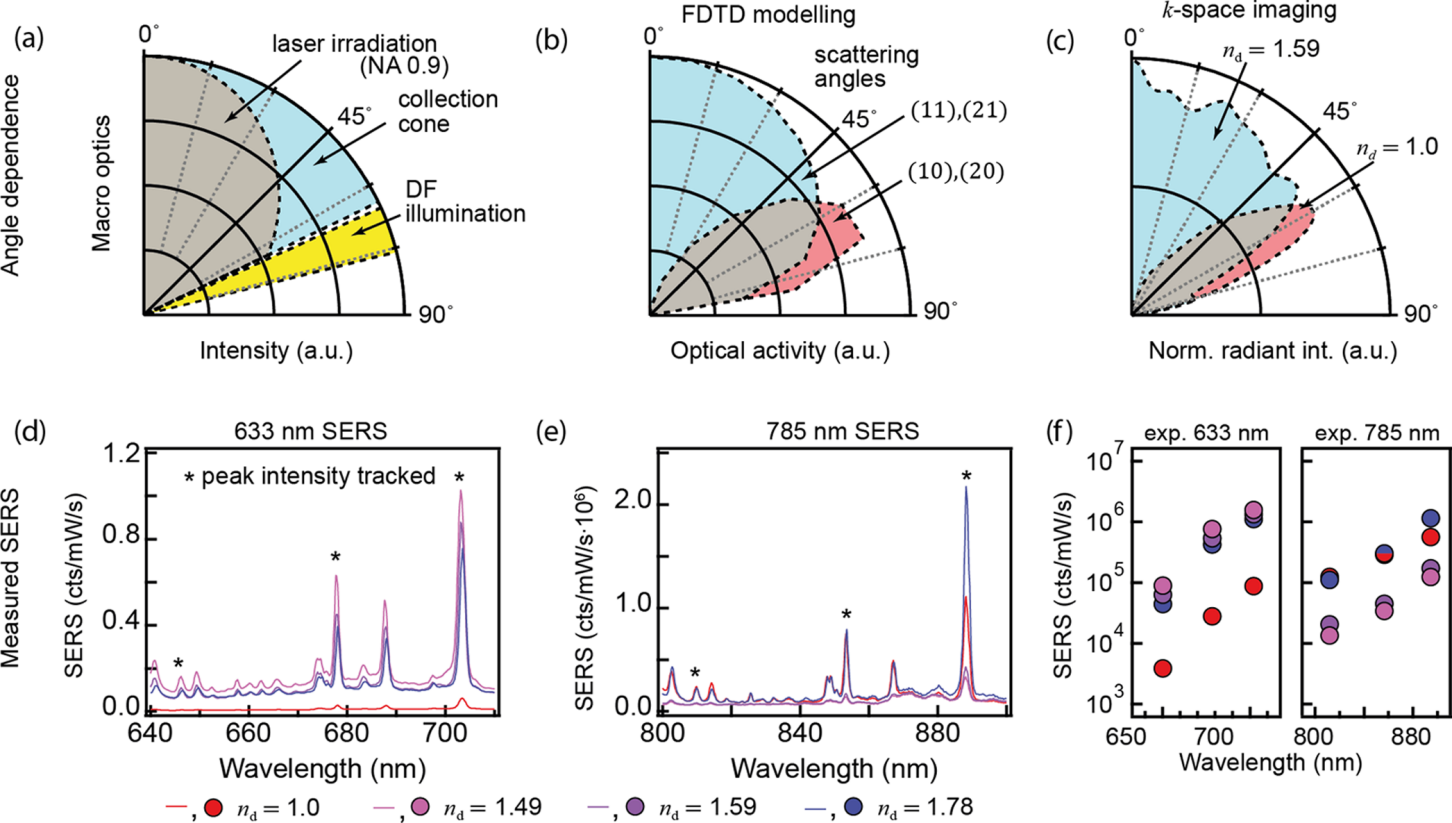
(a) Experimental NA for DF illumination, collection, and laser irradiation cones. (b) Calculated NPoM excitation and radiation cones for both even and odd modes. (c) Experimental *k*-space scattering from NPoM with (*n*_d_ = 1.59, blue) and without (*n*_d_ = 1.0, red) dielectric coating. (d,e) SERS spectra for NPoMs in air or dielectric coatings from *n*_d_ = 1.49–1.78, as well as colloidally grown individual nanolenses (*n*_d_ = 1.49), using (d) 633 and (e) 785 nm lasers. (f) Extracted peak intensities (peaks * in d,e) for each NPoM geometry at 633 nm/785 nm pumping showing improved performance.

Modeling the scattering from different incident angles (Figure 5) shows that even NPoM modes (which dominate for *n* = 1.0) only accept incident light above 45°, whereas odd modes couple to incident light at angles from 0 to 60° (Figure 5b). Increasing the efficiency of the latter modes is thus critical as most incident light arrives at angles <45°, even for a high numerical apertures (NA) illumination, as illustrated for a collimated Gaussian beam over-filling the back-aperture of a 0.9 NA objective (Figure 5a, “laser irradiation”). The angular scattering of DF light from NPoMs is experimentally measured using *k*-space imaging on *n*_d_ = 1.0 and 1.59 samples (Figure 5c, see Supporting Information Note S3, Figure S4 for details). At *n*_d_ = 1, NPoMs scatter near 60° as predicted, while *n*_d_ = 1.59 coated NPoMs scatter over a wide angular range between 0 and 55°. This confirms that odd modes dominate emission when NPoMs are embedded in higher refractive index surroundings.

These benefits from retardation matching can provide significant performance improvements in nonlinear processes from plasmonic nanogap constructs. To demonstrate this, surface-enhanced Raman spectroscopy (SERS) spectra are recorded from the BPT gap molecule using 633 and 785 nm lasers for each refractive index, normalized to the laser power, and corrected for the instrument response^35^ (Figure 5d,e). For 633 nm, embedding gives up to 20× SERS increase, with comparable performance for refractive indices *n*_d_ = 1.49, 1.59, 1.78. Apart from this enhancement, the SERS spectra are nearly identical, showing no additional signal components from the embedding dielectric film (since the SERS originates from the strong hotspot inside the gap, Figure 1a).

Extracting amplitudes of three SERS peaks (*) for each refractive index isolates the vibrational signals from changes in background and noise and demonstrates the clear enhancements from embedding at every spectral position (Figure 5f). For *n*_d_ = 1, the highest SERS signals are collected for 785 nm excitation, as expected from the strong (10) mode at 810 nm. However, when the surrounding refractive index is increased to *n*_d_ = 1.49, 633 nm SERS signals increase by >12×, but SERS signals from 785 nm excitation drop since the (10) mode shifts out of resonance for 1.4 < *n*_d_ < 1.6. The latter SERS intensity recovers for *n*_d_ = 1.78, when higher-order modes shift into resonance, with 633 nm SERS further increasing to 23× (Figure 5f). Note that a continuous increase is observed in SERS intensity from *n*_d_ = 1.49 to 1.59 (see Figure 3), even though there is an anomalous drop in DF intensity, suggesting that this latter arises from high angle excitation (for DF spectroscopy).

To gain a better insight into these enhancements and distinguish them from wavelength tuning, a simple nanocavity model is devised. Simulations of scattering spectra and near-field enhancements give the parameters *E*^2^, *V*, *Q* for the near-field intensity enhancement, mode volume, and quality factor of each mode respectively (Supporting Information note 4). The mode coupling efficiency *C*_lm_ into the nanocavity is then estimated as (see ref (8))
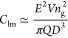
1

This extracted coupling rate for the (10) mode increases by 50% as the NPoM is progressively covered with an *n*_d_ = 1.6 coating (Supporting Information). The coupling of the higher-order odd modes (11), (21) increases by >200% (see Supporting Information). This again shows that the initially dark modes become much brighter, through the increased retardation of light through the dielectric layer. The effective optical path between the AuNP top and bottom approaches λ/2, which then matches the magnetic-type coupling of (*l*1) as clearly seen by the phase difference across the AuNP in Figure 2e for the (21) mode.

## Conclusions

In summary, we show through both simulation and experiment how tuning of the surrounding refractive index can improve the optical performance of plasmonic nanocavity constructs. Increases in refractive index dramatically improve the acceptance and out-scattering angles of such structures. Experiments and simulations show that this occurs by increasing the optical coupling of the antisymmetric odd modes in the plasmonic nanogaps and give more than 10-fold amplification in signal intensities for the same incident laser power in SERS-sensing applications. These results are more generally applicable to a wide range of nanogap plasmonic structures since the field orientations perpendicular to the metal surfaces are universal, although the details of peak positions and angles will vary for individual constructs as will the antenna mode coupling. This understanding should encourage new strategies to further boost the optical performance of plasmonic nanostructures.

## Methods

### Sample Preparation

All chemicals were ordered from Sigma-Aldrich, unless noted otherwise, and used as received. To prepare the NPoM geometry, an atomically flat (111) silicon wafer was coated with a 100 nm Au film using a Lesker E-beam evaporator at a rate of 0.1 Å/s. Then, 2 μL droplets of a two-part epoxy glue (Epo-Tek 377) were deposited on the Au-coated wafer to attach Si chips of size approximately 5 mm. The epoxy was cured at 150 °C for 2 h, and the wafers were gradually cooled back down to room temperature. The Si chips were peeled off the wafer, exposing a clean, flat Au surface which was transferred to a 1 mM solution of BPT (97%) in ethanol (≥99.5%, absolute) and left overnight. The BPT-coated samples were rinsed with ethanol and blown dry using nitrogen, and 80 nm AuNPs were deposited by resting a 10 μL drop of colloidal suspension (BBI Solutions, OD1, citrate stabilized) on each of the samples for 20 s.

Index *n*_d_ = 1.49: PMMA dissolved in anisole at 2 wt % (commercial PMMA A2 solution by MicroChem), spun at 1500 rpm with 500 rpm/s acceleration. The film thickness is 112 nm. Cauchy coefficients (MicroChem datasheet): *A* = 1.478, *B* = 7.204 × 10^–4^, *C* = −3.478 × 10^–4^.

Index *n*_d_ = 1.59: poly(2-chlorostyrene) dissolved in chloroform at 1 wt %, spun at 4000 rpm with 500 rpm/s acceleration. The film thickness is 119 nm. Cauchy coefficients: *A* = 1.588, *B* = 3.19 × 10^–3^, *C* = 8.2 × 10^–4^.

Index *n*_d_ = 1.79: poly(pentabromophenyl methacrylate) dissolved in anisole at 7 wt %, spun at 2000 rpm with 500 rpm/s acceleration. The film thickness is 101 nm. Cauchy coefficients: *A* = 1.779, *B* = −4.45 × 10^–3^, *C* = 4.79 × 10^–3^.

### NPoM Characterization

DF spectra were collected using an Olympus BX51 microscope, fiber coupled to an Ocean insight QE65Pro spectrometer. In-house particle tracking software was used to identify and characterize individual NPoM geometries, see ref (36) for details. For SERS, spectra were collected using a homebuilt Raman spectroscopy setup consisting of two single frequency diode lasers (633 and 785 nm) and a Triax 320 spectrometer with a 150 L/mm grating paired with a back-illuminated EMCCD. The relative low lines/millimeter grating allows for simultaneous collection of SERS at 633 and 785 nm excitation.

### *K*-Space Imaging and Angle-Resolved DF Scattering Spectroscopy

Individual nanostructures are illuminated with focused incoherent white light at an annular illumination angle of 64–75° with respect to normal incidence. Scattered light at <64° is collected through a DF objective (Olympus 100xBD, NA 0.9). The scattering pattern is determined using the light intensity distribution in the back focal plane of the microscope objective. Single nanostructures are spatially isolated by spatially filtering the magnified real image plane with a pinhole. The back focal plane image is demagnified three times before being imaged on the entrance slit (150 μm wide) of a Triax 320 spectrometer, where a narrow range of the scattering pattern near *k*_x_/*k*_0_ = 0 is filtered and dispersed by grating and collected using an Andor Newton 970 BVF EMCCD (Figure S4). Using an MFP-3D AFM System (Asylum/Oxford Instruments), the flatness of the gold film, polymer films, and polymer-coated NPoMs was characterized (Figure S10), yielding an RMS of 0.30 Å for both bare gold and the polymer film. For the NPoM samples, occasional small (2–9 nm) bumps are observed, which we attribute to the covering of nanoparticles. Larger bumps are also observed, which likely arise from air bubbles or dust.

### FDTD Simulations

Full-wave 3D simulations are performed using Lumerical FDTD solutions. The AuNP is modeled as a truncated sphere (with a facet width of 20 nm) of radius 40 nm on top of an infinite dielectric sheet of the refractive index of *n*_g_ = 1.45 and a gap size of 1.3 nm matching the BPT thickness.^29^ The thickness of the Au slab placed below the BPT layer is infinite to the perfectly matching layer, and the AuNP is embedded into a dielectric film of different heights and refractive index (*n*_d_) as mentioned in the text. The NPoM geometry is illuminated with a plane wave with polarization either perpendicular or parallel to the metal surface to access different sets of modes. For estimating field enhancements, a 2D near-field monitor is placed at the center of the nanogap. To extract the respective field strengths for each different mode, the near-field spectrum at the field maximum is extracted with multipeak fitting for that resonating mode wavelength.

## Data Availability

Data for all figures can be found at DOI:10.17863/CAM.92627.

## Supporting Information Available

Effect of the surrounding refractive index on the near field of gap modes; reproducibility of polymer film-coated samples; DF illumination angles; back focal plane DF scattering imaging and spectroscopy; summary of model and definitions; radiative Purcell; scattering properties of NPoM for different dielectric coating heights; near-field enhancement across the center of the gap; SERS enhancement in NPoM as a function of coating height; and AFM measurements (PDF)
