# Supplementary material for: Boosting Optical Nanocavity Coupling by Retardation Matching to Dark Modes
Source: ACS Photonics. 2023 Jan 11;10(2):493–9. doi: 10.1021/acsphotonics.2c01603 (PMC9936626; doi:10.1021/acsphotonics.2c01603)
Supplement: Supplementary file 1 — ph2c01603_si_001.pdf [file ph2c01603_si_001.pdf]

Supporting Information

Boosting Optical Nanocavity Coupling by Retardation Matching to Dark Modes

Rohit Chikkaraddy[1], Junyang Huang[1], Dean Kos[1], Eoin Elliott[1], Marlous Kamp[1], Chenyang Guo[1], Jeremy J. Baumberg[1*], Bart de Nijs[1*]

[1] NanoPhotonics Centre, Cavendish Laboratory, Department of Physics, JJ Thompson Avenue, University of Cambridge, Cambridge, CB3 0HE, United Kingdom

Supporting information

Supplementary note 1: **Nanogap mode nearfield enhancements with increasing surrounding refractive index.**

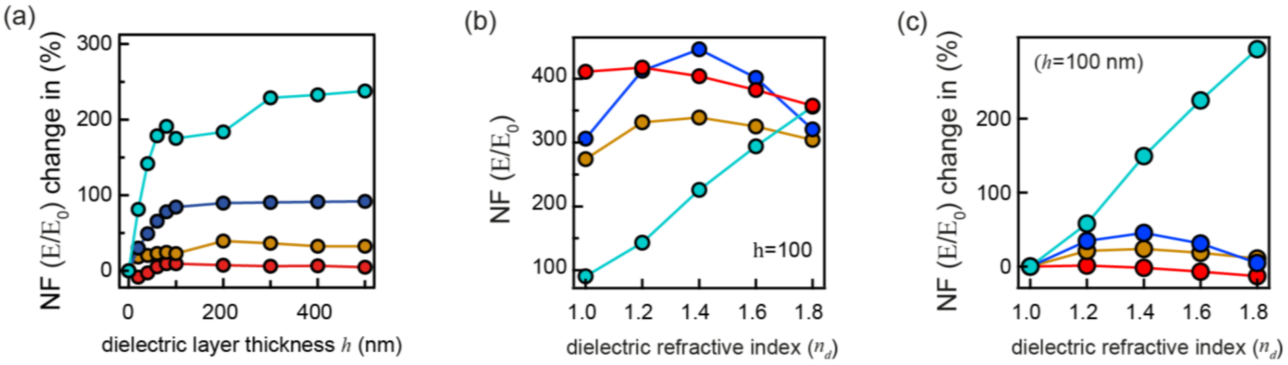

Figure S1: Effect of surrounding refractive index on the nearfield of plasmonic gap modes. a) Percentage enhancement of each mode with increase in h for a high refractive index (n_d=1.5) dielectric coating. b) Nearfield enhancement as a function of the refractive index of the embedding dielectric medium (n_d). c) Relative nearfield enhancements in percentage vs refractive index of the embedding dielectric medium (n_d).

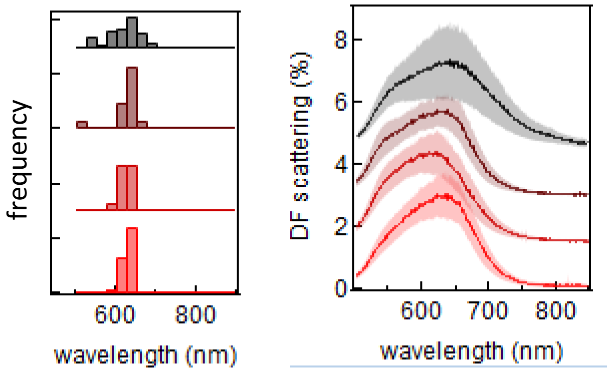

Figure S2: Reproducibility of polymer coated samples. Three additional repeats of the PMMA polymer coating were performed and darkfield scattering spectra from 63, 763, 203 particles were collected. Binning the main peak position shows a histogram with a clear reoccurring peak position between 625-650nm. The scattering intensities for the average spectra show excellent reproducibility (2.9, 2.9, 2.7, 2.9%).

Supplementary note 2: **Illumination geometries**

To determine the illumination geometry of the objective used, the DF illumination was visualised on a white sheet of paper placed perpendicular to the image plane. The angle of DF illumination was measured to be 75° (Figure S3).

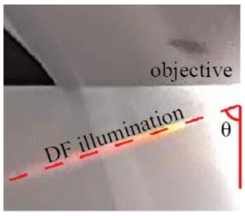

Figure S3: Darkfield illumination angle (θ). Measured for an MPLFLN-BD 0.9NA 100x Olympus objective, visualized on a white piece of paper placed perpendicular to the focal plane.

Supplementary note 3: **Angle dependent scattering spectroscopy**

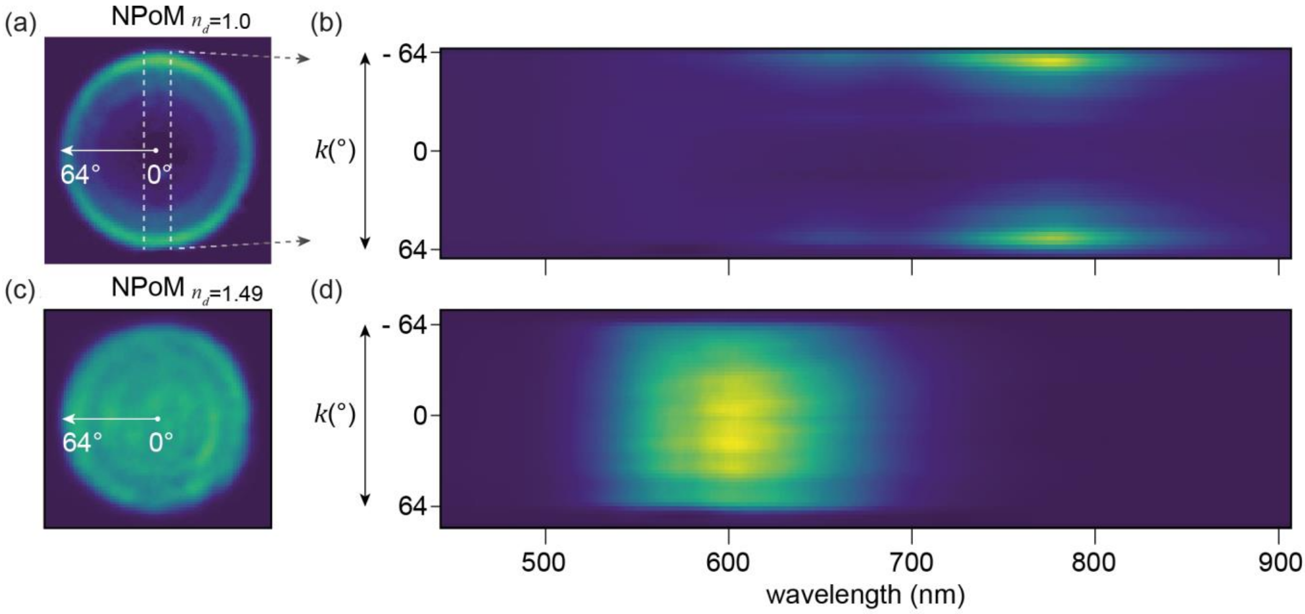

Figure S4: Back focal plane (BFP) dark field scattering images captured from a single NPoM (a) with and (c) without refractive index coating (n_d=1.0, n_d=1.49 respectively) using a NA=0.9 dark field objective. (b,d) Corresponding angle-resolved DF scattering spectra captured by filtering a wavevector range of the BFP image near k_x/k_0=0 (dashed lines in a) and dispersed by a 150 lines/mm grating.

Supplementary note 4: **Analysis of Scattering and Near-field in NPoMs**

FDTD simulations give the spectrum of scattering $S(\lambda)$ and the near-field enhancement $\mathrm{E}(\lambda) = E_i/E_0$. From this the resonant peak λ_n can be extracted for each mode ($n = 10{,}20{,}11, \ldots$) which is similar in both S and E. From FDTD, then 3 parameters can be extracted $\{S_{pk}, \mathrm{E}_{pk}, \Delta\lambda\}$ for each mode (Figure S5).

Scattering S (in units m^2) comes from scattered power (in W) = S x incident intensity (W/m^2). The scattered power per cycle (period λ/c) is the total energy in the cavity (U) x fraction radiated per cycle

(l_r). The energy in the cavity is $U = V\frac{1}{2}\varepsilon_g\varepsilon_0 E_i^2$ for cavity volume V for each mode, while the incident intensity is $\frac{1}{2}c\varepsilon_0 E_0^2$. Hence

$$Ul_r\frac{c}{\lambda} = S\,\tfrac{1}{2}c\varepsilon_0 E_0^2$$

or rearranging

$$l_r = \frac{S}{\mathrm{E}^2}\frac{\lambda}{V\varepsilon_g}$$

which is dimensionless as required. We use the peak values on resonance for S,E.

To obtain the mode volumes, the nearfield profile across the gap is used, $\mathrm{E}(\lambda_n, x)$. The mode volume is defined as

$$V = \frac{\int \mathrm{E}^2 dV}{\max(E^2)} = \frac{2\pi d \int \mathrm{E}^2 r dr}{\max(E^2)}$$

with an extra factor of ½ for the odd (11),($21)$ modes which have an additional sin(φ) dependence. Note that the volume so far omits out the field which penetrates slightly into the metal facet on either side of the nanocavity, as well as the much weaker field around the rest of the NP. The decay length δ of light into the metal follows from the resonant wavevector $k_{\parallel}w = 2\alpha$ for facet diameter w and Bessel zero α, since $k_{\perp}{\sim}ik_{\parallel}$ and $\delta = k_{\perp}^{-1} = w/2\alpha$ ~5 nm, thus larger than d. The extra metallic volume contribution is then $2.\delta.\pi\left(\frac{w^2}{4}\right)$ but the field is smaller by $\frac{\varepsilon_m}{\varepsilon_g} = \frac{w}{d\alpha}$, which gives $V_m = \varepsilon_m\left(\frac{d\alpha}{w}\right)^2\frac{w^3\pi}{4\alpha} = \varepsilon_g w^2 d\pi/4 \sim$ 850 nm^3, which is twice as large as the original gap volume. As a result, the total volume is three times the original integration above. Indeed to match previous estimates for $l_r{\sim}0.6$ at h=0 (from COMSOL calculations), a volume roughly double the integration volume is required.

Note that the integrated scattered flux S is derived from the peak scattered flux S_{pk} (in a particular direction). This is different for the normally radiating modes ($n1$) and the high angle modes ($n0$), and is evaluated using calculated mode patterns as shown in ref[1].

The total Q-factor can be extracted from the linewiths, $Q_t = \lambda_n/\Delta\lambda_n$, but we note that there is considerable confusion in the literature about whether an extra prefactor of 2π is included. To reproduce the known values of $l_r = 0.7$ at h=0, the appropriate formula is $Q_t = 2\lambda_n/\Delta\lambda_n$. Since $Q = 2\pi/l$, we extract $l_t = 2\pi/Q_t$ and

$$l_{nr} = l_t - l_r$$

so that the radiative efficiency for each mode can be defined as

$$\eta_S = l_r/(l_r + l_{nr})$$

Finally, we obtain the field enhancement inside the nanogap. In ref[2], the antenna mode coupled energy was estimated from the polarizability of the NP to give the energy coupled into the nanogap

$$\tfrac{1}{2}\varepsilon_0\varepsilon_g E_\mathrm{i}^2\,\mathrm{V} = Q_t.2\pi\varepsilon_0 R^3 \beta E_0^2$$

where $\beta{\sim}2$ is the spectral enhancement here, which gives

$$\mathrm{E}^2 = \frac{E_\mathrm{i}^2}{E_0^2} = \zeta\frac{Q_t}{V}\frac{8\pi}{\varepsilon_g}R^3$$

with an extra coupling factor ζ included for how well each mode couples, and which describes the effective antenna cross section.

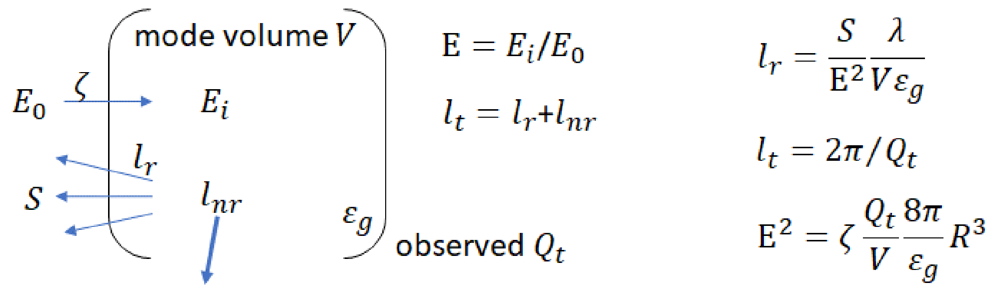

Figure S5: Summary of model and definitions

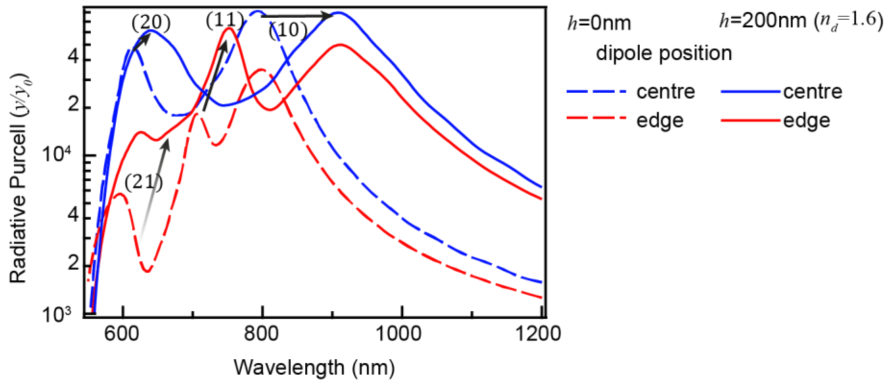

Figure S6: Radiative Purcell measured at the centre and edge of the nanocavity for both for no dielectric layer and a layer height of h=200nm, from which the change in scattering intensity is extracted for each mode.

As noted above, we scale V and Q to match the calculated $l_{r,nr}(h = 0)$ for (10) for coating refractive index $n_d = 1.6$. The extracted parameters for each mode are plotted below (Figure S5), except for (21) as the initial uncoated intensity of this mode is too weak to be extracted.

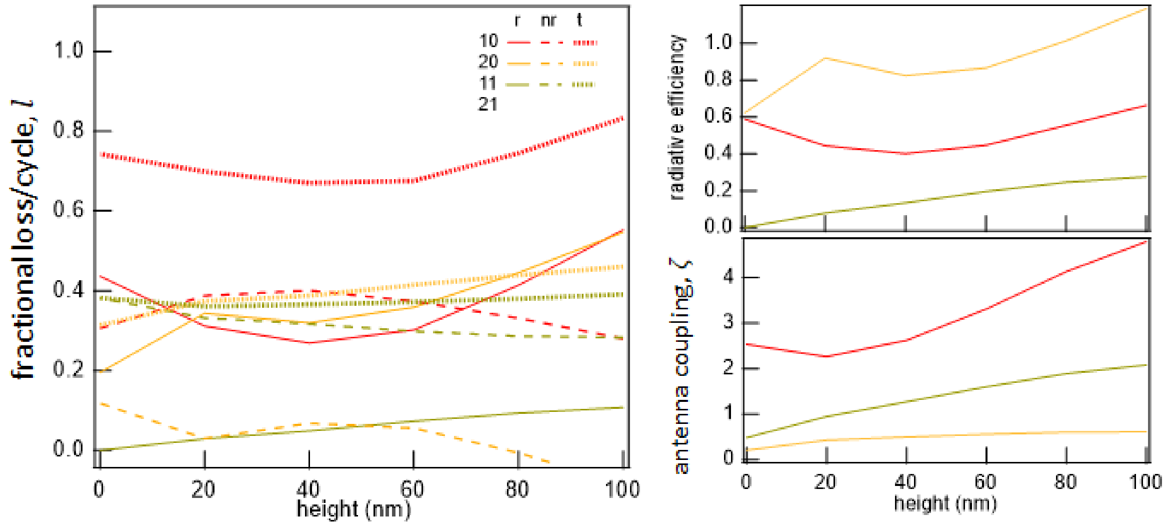

Figure S7: Scattering properties of NPoM for different dielectric coating heights. (a) Fractional loss rate per cycle of radiative (r), non-radiative (nr) and total (t) components *vs* dielectric coating height, for each mode. (b,c) Corresponding radiative efficiency and coupling effectiveness (antenna cross section).

This gives an understanding for how the dielectric layer coating acts (Figure S7) to give:

- increased E with dielectric layer height from the increased antenna cross section, strongest for (20, 11) modes,
- strong increase of (11) in near-field and scattering due to increases in both in/out coupling (ζ,η),
- (20) radiating strongly, but has poor in-coupling hence only appears weakly in S for low h,

- larger linewidth of (20) which comes as both radiative and non-radiative emission is faster,
- a linewidth of (11) dominated by non-radiative components, while (20) is dominated by radiative components.

From this it is clear that the dielectric layer helps in-coupling, but particularly for higher order modes. The dielectric layer also drastically increases the outcoupling for the (11) mode, which can be intuitively considered as helping plasmons escape out around the edge of the nanocavity onto the surface of the NP.

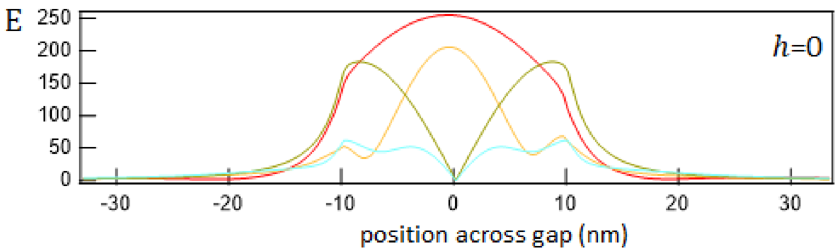

Figure S8: Near-field enhancement across the centre of the gap, for no dielectric coating.

Using this model the SERS enhancements can be predicted, since the enhancement (ignoring input/output wavelength differences) is $\eta \mathrm{E}^4$.[3] The nanocavity centre favours 'even' modes due to the central antinode, but we integrate over all molecules overlapping the modes to get the predicted SERS emission $= \eta \mathrm{E}^4 V/(\sigma^2 d)$ where σ is the size of a molecule (Figure S9).

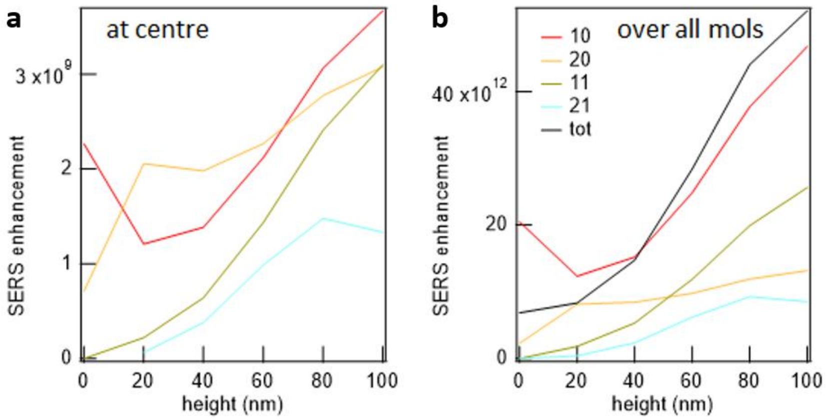

Figure S9: SERS enhancement in NPoM as a function of dielectric coating height. a) scaling at centre position, b) and integrated over all molecules.

This model shows that, independent from wavelength tuning, as a result of the retardation matching alone the local refractive index (n_d) can provide an increase in SERS of nearly 10x when fully embedded (here calculated for n_d=1.6).

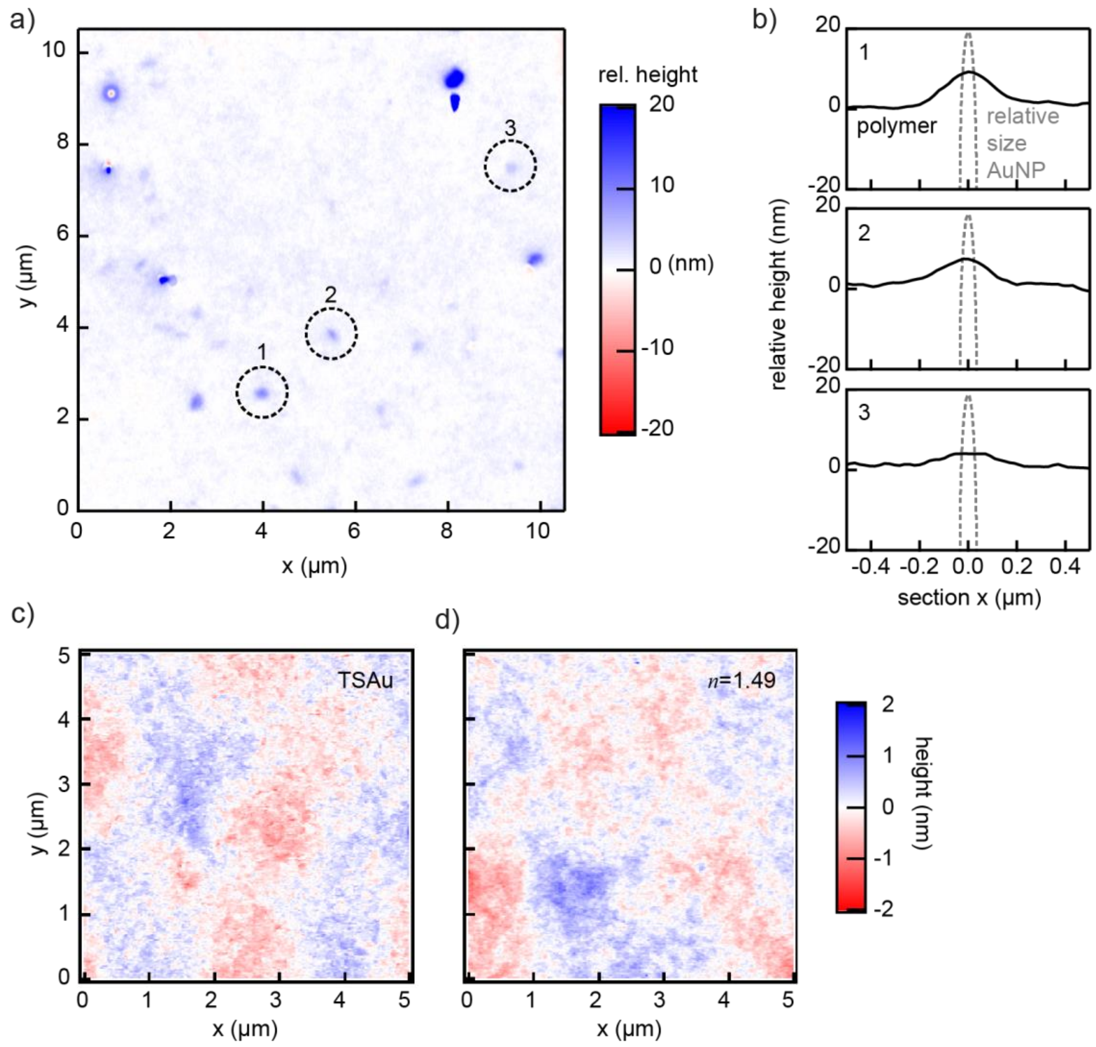

Figure S10: AFM measurements a,b) Polymer-coated (n_d=1.49) NPoMs showing residual 2-9 nm local deformations of the surface normal as a result of the underlying nanoparticle inside the embedding film of nominal height 119 nm (measured by ellipsometry, see methods). c) AFM map of bare template-stripped gold showing long-range height variations ~1nm and a roughness of RMS=3.0Å. d) AFM map of polymer-coated gold without NPoMs showing little change from the bare gold and an RMS of 3.4 Å.

References

(1) Kongsuwan, N.; Demetriadou, A.; Horton, M.; Chikkaraddy, R.; Baumberg, J. J.; Hess, O. Plasmonic Nanocavity Modes: From Near-Field to Far-Field Radiation. *ACS Photonics* **2020**, *7* (2), 463–471. https://doi.org/10.1021/acsphotonics.9b01445.

(2) Baumberg, J. J.; Aizpurua, J.; Mikkelsen, M. H.; Smith, D. R. Extreme Nanophotonics from Ultrathin Metallic Gaps. *Nat. Mater.* **2019**, *18* (7), 668–678. https://doi.org/10.1038/s41563-019-0290-y.

(3) Le Ru, E. C.; Etchegoin, P. G. Rigorous Justification of the |E|4 Enhancement Factor in Surface Enhanced Raman Spectroscopy. *Chemical Physics Letters* **2006**, *423* (1), 63–66. https://doi.org/10.1016/j.cplett.2006.03.042.

(4) Nijs, B. de; Bowman, R. W.; Herrmann, L. O.; Benz, F.; Barrow, S. J.; Mertens, J.; Sigle, D. O.; Chikkaraddy, R.; Eiden, A.; Ferrari, A.; Scherman, O. A.; Baumberg, J. J. Unfolding the Contents of Sub-Nm Plasmonic Gaps Using Normalising Plasmon Resonance Spectroscopy. *Faraday Discuss.* **2015**, *178* (0), 185–193. https://doi.org/10.1039/C4FD00195H.

(5) Kamp, M.; Nijs, B. de; Kongsuwan, N.; Saba, M.; Chikkaraddy, R.; Readman, C. A.; Deacon, W. M.; Griffiths, J.; Barrow, S. J.; Ojambati, O. S.; Wright, D.; Huang, J.; Hess, O.; Scherman, O. A.; Baumberg, J. J. Cascaded Nanooptics to Probe Microsecond Atomic-Scale Phenomena. *PNAS* **2020**, *117* (26), 14819–14826. https://doi.org/10.1073/pnas.1920091117.

(6) Benz, F.; Tserkezis, C.; Herrmann, L. O.; de Nijs, B.; Sanders, A.; Sigle, D. O.; Pukenas, L.; Evans, S. D.; Aizpurua, J.; Baumberg, J. J. Nanooptics of Molecular-Shunted Plasmonic Nanojunctions. *Nano Lett.* **2015**, *15* (1), 669–674. https://doi.org/10.1021/nl5041786.
